# The Signaling Pathways Involved in Ovarian Follicle Development

**DOI:** 10.3389/fphys.2021.730196

**Published:** 2021-09-20

**Authors:** Liyuan Li, Xiaojin Shi, Yun Shi, Zhao Wang

**Affiliations:** ^1^Protein Science Key Laboratory of the Ministry of Education, School of Pharmaceutical Sciences, Tsinghua University, Beijing, China; ^2^Tsinghua-Peking Center for Life Sciences, Beijing, China; ^3^Dongzhimen Hospital, Beijing University of Chinese Medicine, Beijing, China

**Keywords:** follicular development, PI3K-AKT signaling, WNT signaling, insulin signaling, Notch signaling, Hedgehog signaling

## Abstract

The follicle is the functional unit of the ovary, which is composed of three types of cells: oocytes, granulosa cells, and theca cells. Ovarian follicle development and the subsequent ovulation process are coordinated by highly complex interplay between endocrine, paracrine, and autocrine signals, which coordinate steroidogenesis and gametogenesis. Follicle development is regulated mainly by three organs, the hypothalamus, anterior pituitary, and gonad, which make up the hypothalamic-pituitary-gonadal axis. Steroid hormones and their receptors play pivotal roles in follicle development and participate in a series of classical signaling pathways. In this review, we summarize and compare the role of classical signaling pathways, such as the WNT, insulin, Notch, and Hedgehog pathways, in ovarian follicle development and the underlying regulatory mechanism. We have also found that these four signaling pathways all interact with FOXO3, a transcription factor that is widely known to be under control of the PI3K/AKT signaling pathway and has been implicated as a major signaling pathway in the regulation of dormancy and initial follicular activation in the ovary. Although some of these interactions with FOXO3 have not been verified in ovarian follicle cells, there is a high possibility that FOXO3 plays a core role in follicular development and is regulated by classical signaling pathways. In this review, we present these signaling pathways from a comprehensive perspective to obtain a better understanding of the follicular development process.

## Introduction

The ovary is an important organ in the reproductive system of female mammals. The main functions of the ovary are to produce and periodically release oocytes and secrete steroid hormones. Ovarian follicles are composed of different numbers and types of cells, which provide an appropriate environment for oocyte development (Gougeon, 1996). The menstrual cycle of the ovary can be divided into three phases: the follicular phase, ovulation phase, and luteal phase. Ovarian follicles develop under the effects of hormones. During the follicular phase, one or two follicles become dominant and subsequently release an oocyte under the stimulation of luteinizing hormone. The remaining part of the follicle becomes a corpus luteum (CL), which can produce progesterone for luteal phase and pregnancy (Xie et al., 2017). Follicles during the folliculogenesis process can be divided into three stages: primordial follicles (PmF), growing follicles, and Graafian follicles (GF). Growing follicles can be further divided into primary follicles (PrF) and secondary follicles (SF). Follicles that cannot ovulate are called atretic follicles (AF) (Eppig and O’Brien, 1996). Ovarian size is related to the woman’s age and spawning cycle, which is closely related to the types and numbers of follicles (Li and Wang, 2018). The follicle is the functional unit of the ovary; therefore, ovarian development is essentially follicular development.

In the fourth month of embryonic development, the ovary contains approximately 6–7 million eggs coated with monolayer granulocytes, an area also known as the primitive follicular area (Block, 1951; Baker, 1963). In the later stages of embryonic development, primordial follicles are rapidly lost due to apoptosis. At birth, fewer than 2 million primordial follicles are present (Markstrom et al., 2002). After the infant is born, the rate of follicular apoptosis gradually decreases, and the number of egg cells is approximately 300,000–400,000 until menarche (Block, 1951). During a woman’s reproductive years, the number of primordial follicles continues to decline, and this rate of decline accelerates until menopause, when the number falls below 1,000, and the ovary gradually loses its normal ability to produce and releases oocytes periodically (Faddy and Gosden, 1996; Faddy, 2000). Follicular development is the result of the regulation and interaction of hormones and proteins.

Some diseases, such as polycystic ovary syndrome (PCOS) and premature ovarian failure (POF), can also cause ovarian dysfunction. With the abnormal hormonal support associated with ovarian dysfunction, a number of physiological systems, including cardiovascular health and bone density, are affected (Gosden, 1986; Sherwin, 1998). The molecular mechanism of ovarian development is complicated, and understanding the signaling pathways in ovarian development will be of great value for discovering the causes of ovarian dysfunction. In this article, we discuss classical signaling pathways during follicular development.

### PI3K/AKT/FOXO3 Signaling in Follicle Development

The phosphatidylinositol 3-kinases (PI3K) are a family of enzymes that share the function to phosphorylate the 3-hydroxyl group of phosphoinositides (Cantley, 2002), it is activated by diverse growth factor receptors (GFR) and oncogenes (Fruman et al., 2017). PI3Ks can be divided into three classes, among which Class I PI3Ks were the most studied to play important roles in regulating cell proliferation and survival (Cully et al., 2006; Jiang and Liu, 2009). The serine/threonine kinase AKT/PKB is a growth factor which exists as three isoforms: AKT1, AKT2, and AKT3. It is a key downstream target of PI3K and a central medium for the PI3K pathway (Zhang and Zhang, 2019). The production of the phosphoinositide PI(3,4,5)P3 (PIP3) from PI(4,5)P2 in the plasma membrane is catalyzed by PI3K, resulting in membrane recruitment, phosphorylation, and the activation of AKT (John et al., 2008). Once activated, AKT moves to the nucleus and cytoplasm, where a series of downstream targets containing AKT recognition motif were phosphorylated and activated/suppressed, including GSK3, BAD, TSC2, and FOXOs, thus mediates a variety of metabolic effects (Yao and Cooper, 1995; Manning and Cantley, 2007).

The forkhead box O (FOXO) transcription factors are negatively regulated by PI3K/AKT signaling pathway (Zhang and Zhang, 2019). In mammals, FOXO family consists of FoxO1, FoxO3, FoxO4, and FoxO6 proteins, they express ubiquitously in the body and the four isoforms share a common structural motif named “forkhead box” domain that is responsible for binding to chromatin DNA (Accili and Arden, 2004). FOXO1, FOXO3, and FOXO4 are directly phosphorylated by AKT, leading to nuclear export and transcriptional inhibition (Zhang and Zhang, 2019). The transcriptional activity of FOXOs is regulated by shuttling between the nucleus and the cytoplasm. Upon loss of GFR signaling, dephosphorylation of PIP3 by PTEN leads to reduced AKT activity, FOXO presents in its non-phosphorylated form and was accumulated in the nucleus. In the nucleus, FOXOs mediate transcription of a series of target genes which involves in cell proliferation, cell cycle, apoptosis and so on (Eijkelenboom and Burgering, 2013). Once the PI3K/AKT pathway is activated, FOXO is phosphorylated by AKT and excluded from the nucleus, leading to the suppression of target gene transcriptional activation and the inhibition of cell proliferation (Stefanetti et al., 2018). Some extracellular ligands are considered to be the target genes for the FOXO family, including FasL (the Fas ligand), TRAIL (TNF-related apoptosis-inducing ligand), and TRADD (TNF receptor type 1 associated death domain), and the intracellular apoptotic components such as Bim (bcl-2 interacting mediator of cell death), a pro-apoptotic Bcl-2 family member, and Bcl-6 (Zhang and Zhang, 2019). PI3K-AKT-FOXO signaling is the central pathway controlling growth and metabolism in all cells (Goldbraikh et al., 2020).

Of all the FOXO family proteins, we are most interested in FOXO3 (also called FKHRL1) protein, which was originally known to regulate insulin signaling (Link and Fernandez-Marcos, 2017; Menon and Ghaffari, 2018), yet an increasing number of studies shows that PI3K/AKT/FOXO3 signaling is closely related with ovarian function. During follicle development, PI3K signaling pathway controls primordial follicle activation through FOXO3 (John et al., 2008). FOXO3 is regulated by nucleocytoplasmic shuttling within oocytes. During primordial follicle assembly, FOXO3 is non-phosphorylated and localized to the nucleus, where it acts to suppress the activation of primordial follicles. The activation of PI3K/AKT results in FOXO3 phosphorylation and nuclear export, thereby triggering primordial follicle activation (John et al., 2008). Studies by Pelosi et al. (2013) showed that constitutively active FOXO3 in oocytes can increase ovarian reproductive capacity in mice. Increased follicle numbers and decreased gonadotropin levels were observed in aging FOXO3 transgenic mice compared to wild-type littermates, indicating the maintenance of a greater ovarian reserve. The study also found that FOXO3 expression in transgenic mice produced a younger-looking gene expression profile, while the gene expression profile of ovaries from Foxo3^–/–^ knockout mice appeared more mature than that of WT littermates.

The body level of 2,5-Hexanedione (2,5-HD) was high in some smokers, drinkers, and those workers who use n-hexane, which will lead to the damage of female reproductive system like early menopause, menstrual disorders, and reduced fertility (Hou et al., 2020). In the study conducted by Zeng et al. (2020), a whole ovary culture models were used to observe the effect of 2,5-HD on follicular growth and development. They found that 2,5-HD can upregelate miR-214-3p, which directly targets PI3K and thus disrupts PI3K/AKT/FOXO3 signaling pathway, leading to the inhibition of primordial follicles development (Zeng et al., 2020). PCOS is a common endocrine and metabolic disease that influences ovarian function, which is often accompanied by insulin resistance. A recently study showed that FOXO3 was increased in granulosa cells of PCOS patients. LNK, an important regulator of insulin signaling pathway, can promote granulosa cell apotosis in PCOS via negatively regulating insulin-stimulated PI3K/AKT/FOXO3 pathway (Tan et al., 2021). Another study by Choi et al. (2020) also found that Placenta-derived mesenchymal stem cells (PD-MSCs) transplantation can restore ovarian function and induce ovarian folliculogenesis via the PI3K/AKT/FOXO3 signaling pathway (Choi et al., 2020). Another recent study found that hypomethylated FOXO3 mRNA caused the dysregulation of FOXO3 in luteinized GCs from PCOS patients following controlled ovarian hyperstimulation (Zhang et al., 2020). All these findings emphasize the role of FOXO3 as a guardian of the ovarian follicle pool in mammals and a potential determinant of the onset of menopause (Pelosi et al., 2013).

Previous studies showed that the FOXO3a was closely associated with human longevity. SIRT6, which is a deacetylase, was also shown to be related to human longevity. It was reported that FOXO3a regulated the transcription of SIRT6 by binding and activating nuclear respiratory factor 1 (NRF1) in the mouse. SIRT1, FOXO3a and NRF1 form a complex on the SIRT6 promoter and positively regulates the expression of SIRT6 (Kim et al., 2010), which regulates glycolysis, triglyceride synthesis, and fat metabolism by deacetylating histone H3 lysine 9 in the promoter of many genes involved in these processes. Another study conducted in rats found that obesity accelerates ovarian follicle development and follicle loss in rats, while caloric restriction prolonged ovary lifespan by SIRT1/FOXO3a/NRF1-SIRT6 pathway (Wang et al., 2014), indicating the target of FOXO3 in follicle development.

### WNT Signaling in Follicle Development

**FIGURE 1:**
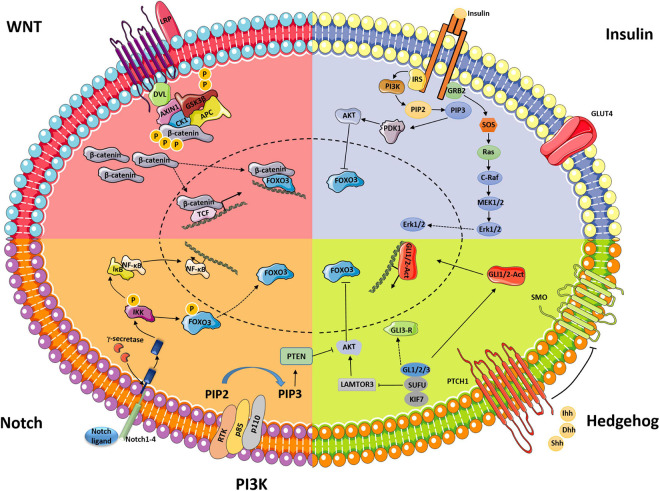
Schematic displaying classical signaling pathways (WNT, insulin, Notch, and Hedgehog) and their interaction with PI3K/AKT-FOXO3 during ovarian follicle development.

WNTs are secreted glycoproteins that regulate multiple signaling pathways through β-catenin-dependent, β-catenin-independent, and WNT/Ca^2+^-related mechanisms (Anastas and Moon, 2013). The canonical WNT-β-catenin signaling pathway, an evolutionarily conserved cell–cell communication system that regulates cell proliferation, differentiation, stem cell renewal, motility, and apoptosis during embryogenesis and adult tissue homeostasis, has been intensively studied (Steinhart and Angers, 2018). In the absence of WNT signaling, β-catenin is phosphorylated at N-terminal sites by the multiprotein degradation complex, which is made of CK1, AXIN1, GSK3β, and APC, and subsequently undergoes ubiquitination and proteosomal degradation within the degradation complex (Hernandez Gifford, 2015). Once WNT binds the frizzled/LPR coreceptor complex, activation of the complex promotes the association of the integrated AXIN1/GSK3/APC degradation complex with the phosphorylated tail of LRP. The degradation complex still binds phosphorylated β-catenin, but ubiquitination by β-TrCP fails to occur. Once phosphorylated β-catenin saturates the degradation complex, newly synthesized β-catenin accumulates in the cytoplasm and subsequently translocates to the nucleus, where it initiates gene transcription (Figure 1; Hernandez Gifford, 2015). A series of studies have been conducted to assess the regulation of WNT signaling in follicle development and formation of the CL (Albert et al., 2002; Minnie et al., 2002; Harwood et al., 2008).

*Wnt2* is expressed in the granulosa cells of rat ovaries at all stages of follicle development (Albert et al., 2002), and the Wnt2 protein expression level is high in preantral and antral follicles (Wang et al., 2010). After treatment with FSH, the mRNA level of *Wnt2* was increased in cultured granulosa cells (Castanon et al., 2012). A study conducted by Wang et al. (2013) demonstrated that WNT2 regulates DNA synthesis in mouse granulosa cells through β-catenin. Knockdown of *Wnt2* by RNAi technology inhibited granulosa cell proliferation. Additionally, overexpression of WNT2 in granulosa cells promoted cell proliferation and increased β-catenin levels in both the cytoplasm and nucleus (Wang et al., 2010). In addition, WNT2 can regulate gap junction signaling in folliculogenesis (Wang et al., 2013).

*Wnt4* is also expressed in murine granulosa cells throughout follicle development (Minnie et al., 2002). After treatment with hCG, the expression of *Wnt4* was increased in rodent granulosa cells (Minnie et al., 2002). Boyer et al. (2010) found that deletion of *Wnt4* in granulosa cells in mice caused subfertile females with fewer healthy antral follicles and smaller ovaries; overexpression of WNT4 in granulosa cells led to the increased expression of β-catenin and its target genes, indicating the important role of WNT4 in follicle maturation. Moreover, *Wnt4* regulates embryonic gonad functions and female gonad development (Yao et al., 2004).

FZ receptors, which interact with WNT proteins to initiate downstream signaling, are expressed at some stages during follicle development, ovulation and luteinization (Hernandez Gifford, 2015). For example, *Fz4* is required for fertility and shows distinct expression in the CL of pregnant mice in rodents. *Fz4-*knockout mice were shown to be capable of producing fertilized oocytes but were sterile because of the failure to form normal corpora lutea (Hsieh et al., 2005).

β-Catenin is the key molecule in the canonical WNT-β-catenin signaling pathway, and deletion of the *Ctnnb1* gene in mice caused infertility due to defects in the oviduct and uterus (Hernandez Gifford et al., 2009). Previous studies also showed that the activation of β-catenin can facilitate FSH-mediated effects in ovarian follicular cells (Hernandez Gifford, 2015), and conditional deletion of β-catenin in mouse granulosa cells resulted in the impaired ability of FSH to stimulate *Cyp19a1* expression, subsequently influencing estradiol production (Hernandez Gifford et al., 2009).

β-Catenin is also linked with the LH-mediated production of progesterone from corpora lutea. In cultured luteal cells, LH stimulation resulted in increased levels of active β-catenin by phosphorylating and inhibiting the function of GSK3 (Lynn et al., 2009). Active β-catenin interacts with the promoter of the *StAR* gene and increases the mRNA expression level of StAR (Hernandez Gifford, 2015). STAR is an important protein that regulates progesterone synthesis by enhancing the conversion of cholesterol into pregnenolone.

Li et al. (2014) discovered that Wnt/β-catenin signaling promotes granulosa cell apoptosis and represses granulosa cell proliferation through the activation of Foxo3a and its downstream target genes. After treatment with LiCl or Wnt3a, which are Wnt/β-catenin signaling activators, the phosphorylated FOXO3a level was significantly decreased. In contrast, FOXO3a phosphorylation was increased by treatment with the Wnt/β-catenin signaling inhibitor IWR-1. These results suggest that FOXO3a is also a downstream effector of Wnt/β-catenin signaling (Li et al., 2014).

### Insulin Signaling in Follicle Development

Insulin was initially characterized for its role in regulating glucose, lipid, and energy homeostasis in muscle, the liver, and adipose tissue (Saltiel and Kahn, 2001). Insulin signaling interacts with two closely related tyrosine kinase receptors (Boucher et al., 2014), and the initiation of signaling leads to the phosphorylation and activation of enzymes that control many aspects of metabolism and growth in a cascade (Boucher et al., 2014).

Insulin signaling is of great significance for female reproductive health. Clinical data have shown that hypoinsulinemia and hyperinsulinemia are associated with significant alterations in ovarian function (Chang et al., 2005; Diamanti-Kandarakis and Dunaif, 2012). The insulin signaling pathway contains different regulation nodes, which ensures proper signal duration and intensity. Insulin has gonadotrophic effects in the ovary, which are mediated by interactions between the respective signaling pathways at critical nodes, such as MAPK and AKT (Figure 1; Taniguchi et al., 2006).

Insulin regulates folliculogenesis in the later stages of oocyte development. In cultured mammalian cells, insulin stimulation was shown to promote oocyte growth by increasing the number of gonadotropin receptors or increasing the sensitivity and binding ability of LH to receptors (Das and Arur, 2017). Insulin can also work synergistically with FSH to promote the differentiation and proliferation of human ovarian theca-interstitial cells (Duleba et al., 1997). Insulin could stimulate both steroidogenesis and cell proliferation in cultured granulosa and theca calls (Saltiel and Kahn, 2001). Moreover, insulin regulates the production of human ovarian androgens, and a study by Bergh et al. (1993) provided evidence of androgen secretion in cultured human theca cells, which are substrates for the synthesis of E2 in granulosa cells. These results indicate that insulin plays an indirect role in the formation of early follicles. Another *in vitro* study carried out in human and animal primate follicles suggested that insulin acts as a survival factor, since the number of atretic follicles decreases when insulin levels increase, leading to an overall increase in the number of living follicles (Henna et al., 2000; Xu et al., 2010). *In vivo* studies conducted by Peluso et al. (1991) in rat ovaries demonstrated that insulin stimulates mitogenic activity and simultaneously suppresses estradiol secretion (Peluso et al., 1991). Insulin receptor (IR) is expressed in ovarian granulosa and theca cells from different species, such as humans, rats and bovines. However, recent studies have shown that the insulin receptor may not be required for oocyte growth since deletion of IR did not influence oocyte development or normal estrous cyclicity (Pitetti et al., 2009).

The FOXO protein act as a nuclear transcription factor and mediates the inhibitory effect of insulin or insulin-like growth factor (IGF-1) on key functions in different pathways (Accili and Arden, 2004; Lee and Dong, 2017). In response to increased insulin (or IGF-1) signaling, the FOXO protein undergo insulin-mediated phosphorylation and translocation from the nucleus to the cytoplasm, resulting in the inhibition of target gene expression (Accili and Arden, 2004; Lee and Dong, 2017). FOXO3 is also a direct target of IκB kinase, and FOXO3 phosphorylation by IκB regulates several cytokine-dependent pathways (Accili and Arden, 2004).

In a recent study, researchers found that the expression level of LNK, a regulator of insulin signaling pathway, was elevated in ovaries of insulin resistant PCOS patients compared with the non-PCOS group, and the overexpression of LNK in ovarian granulosa cell line inhibited insulin induced AKT activation (Fraser et al., 2012). Studies by Tan et al. (2021) found that LNK promotes granulosa cell apoptosis in PCOS via negatively regulating insulin-stimulated AKT-FOXO3 pathway (Tan et al., 2021), demonstrating that insulin signaling influences ovarian granulosa cell proliferation and differentiation through FOXO3 phosphorylation.

### Notch Signaling in Follicle Development

Notch signaling is an evolutionarily conserved pathway in a number of cellular processes, including cell proliferation, cell differentiation, migration, adhesion, and apoptosis (Vanorny et al., 2014; Liu et al., 2019). Notch signaling involves the interaction of one of four Notch receptors (Notch1–Notch4) with one of five Notch ligands (delta-like1, delta-like3, delta-like3, Jagged1, and Jagged2) in mammals (Vanorny et al., 2014). The activation of Notch signaling involves proteolytic cleavages at three sites. Through juxtacrine signaling, Notch ligands bind Notch receptors in the extracellular domain, which causes a conformational change in the Notch receptor and initiates sequential proteolytic cleavage at the juxtamembrane region of the receptor, leading to release of the soluble intracellular domain of Notch (IC-Notch). Through two nuclear localization sequences (NLSs), IC-Notch translocates into the nucleus (Figure 1; Pitetti et al., 2009).

The vascular system provides nutrition, oxygen and hormone support for ovarian follicles and the CL (Robinson et al., 2009); as a result, establishment and continuous remodeling of the vascular system are pivotal to ovarian function. Previous studies have shown that the inhibition of angiogenesis leads to decreased follicle growth and ovulation interruption, which seriously affects the occurrence and development of the CL. Increased thecal vascularity is necessary to maintain the function of the follicles, while decreased thecal vascularity is an important component of follicular atresia (Xie et al., 2017).

Notch proteins and ligands have been detected in the rodent ovary, and studies have shown that Notch receptors and ligands are expressed in a subset of ovarian vessels. For example, Notch 1 was found to be expressed in the endothelium of the theca layer in the follicular-phase ovary, in endothelial cells from the neovasculature of the corpora lutea, and in mature vessels of the theca layer in the luteal phase (Xie et al., 2017). Notch2, Notch3, and Jagged 2 are expressed in the granulosa cells of developing follicles. Dll4 is primarily expressed on endothelial cells at the tip of new vessels (Xie et al., 2017).

Studies have shown that Notch is involved in the angiogenesis process. Dll4 may be a target molecule for ovarian angiogenesis therapy since a decrease in Dll4 *in vivo* in the primate ovary led to increased luteal angiogenesis and microvascular density (Fraser et al., 2012). Notch2 is a key member of Notch signaling and expressed at high levels in theca and granulosa cells of ovarian follicles (Jing et al., 2017). Furthermore, constitutive Notch signaling in adult transgenic mice inhibited bFGF-induced angiogenesis and inhibited follicular development (Xie et al., 2017).

After treatment with the Notch signaling inhibitor DAPT, the number of granulosa cells decreased, and estradiol levels also decreased; furthermore, the expression levels of genes related to the cell cycle and apoptosis decreased (Jing et al., 2017). The *in vitro* treatment of cultured follicles with Notch signaling inhibitors resulted in the complete termination of follicle development, granulosa cell detachment, and oocytes degeneration with cytoplasm condensation (Li et al., 2014). Further studies by Liu et al. (2019) revealed that the proliferation of granulosa cells is dependent on Notch signaling.

Previous studies have shown that in skeletal muscle stem cells (stem cells), FOXO3 regulates the expression of NOTCH1 and NOTCH3 receptors. Conditional deletion of FOXO3 downregulated Notch signaling, leading to impairment of the self-renewal ability of SCs, while overexpression of the Notch intracellular domain (NICD) could rescue this self-renewal deficit (Gopinath et al., 2014). NF-κB and Notch signaling are interconnected under both physiological and pathological conditions (Ferrandino et al., 2018). Previous studies found cross-talk between the NF-κB activation pathway and FOXO3 (Lin et al., 2004). The two kinases involved in NF-κB activation, IKKα and IKKβ, can phosphorylate and activate FOXO3 (Hu et al., 2004). FOXO3 can antagonize NF-κB and regulate cytokine production (Lin et al., 2004). To date, although there have been no related scientific reports, there is a strong potential that Notch signaling regulates follicular development through interactions with FOXO3.

### Hedgehog Signaling in Follicle Development

Hedgehog (Hh) signaling is an evolutionarily conserved signaling pathway that regulates embryonic development as well as many essential tissue and cellular properties, such as cell proliferation, differentiation, and survival. Disruption of the Hh pathway results in serious disease (King et al., 2008). The Hh signaling pathway consists of three ligands, Indian, sonic, and desert Hh (Ihh, Shh, and Dhh, respectively); the membrane receptor patched (PTCH1); and the transmembrane signal transducer protein smoothened (SMO) (Figure 1; Ren et al., 2012).

In the absence of ligand binding, the membrane receptor PTCH1 keeps SMO in an inactive state. Once a ligand binds PTCH1, its inhibition of SMO is unlocked, and signal transduction occurs through the downstream transcription factors GLI1, GLI2, and GLI3 (Huangfu and Anderson, 2006).

Follicle development requires communication between oocytes, granulosa cells, and theca cells (Mark et al., 2005). The granulosa cells in growing follicles act as a source of hedgehog signaling since the expression of Ihh and Dhh mRNA begins at the primary follicle stage. Hh target genes, such as *Ptch1* and *Gli1*, were found to be expressed in surrounding theca cells, and this expression was inhibited by treatment with the Hh signaling antagonist cyclopamine. The dramatic loss of Hh and induced target gene expression was found to be synchronized in periovulatory follicles, demonstrating the role of Hh signaling in communication between granulosa cells and developing theca cells (Mark et al., 2005).

In a study conducted by Yi Ren et al., an *Amhr2*^cre/+^*SmoM2* mouse model in which a dominant active allele of Hh SMO known as SMOM2 was conditionally expressed in the ovary was made. The overactivation of Hh signaling caused anovulation, associated with a lack of smooth muscle in developing follicular theca cells (Ren et al., 2009). The authors’ subsequent study demonstrated that the overactivation of HH signaling early in life in mice alters gene expression and vascular development, which are associated with the lifetime development of anovulatory follicles, during which the follicular vesicles do not mature properly (Ren et al., 2012).

Other factors that influence oocyte production are the number and status of follicle stem cells (FSCs). FSCs are exquisitely responsive to diet-induced signals such as Hh and insulin signaling. Constitutive Hh signaling drives FSC loss and premature sterility by inducing autophagy in FSCs through a Ptc-dependent, Smo-independent mechanism. During the senescence process, Hh-dependent autophagy increases, which triggers FSC loss and leads to reproductive stagnation (Singh et al., 2018).

The late endosomal LAMTOR complex is regarded as a convergence point for the RAF/MEK/ERK and PI3K/AKT/mTOR signaling pathways (De Araujo et al., 2013). A recent study by Klein et al. (2019) also found that LAMTOR3 physically interacts with SUFU and activates mTOR. Deletion of PTCH1 and SUFU caused the activation of PI3K/AKT/mTOR signaling, and the loss of SUFU liberated LAMTOR3 from inhibition and allowed it to activate AKT by increasing the phosphorylation of pAKT473 and pAKT308 (Klein et al., 2019). AKT is the upstream regulator of FOXO3, and the activation of AKT may subsequently lead to changes in FOXO3 phosphorylation.

## Conclusion

Ovarian follicle development and the subsequent ovulation process are coordinated by highly complex interplay between signaling pathways. In this review, we summarize several classical signaling pathways (WNT, insulin, Notch, and Hedgehog signaling) and their functions in follicular development. The ovary is composed of three types of cells: oocytes, granulosa cells, and theca cells. Granulosa cells play an essential role in follicular development; they synthesize and secrete mucopolysaccharides, forming a zona pellucida around the oocyte. The cell membrane protuberances of granulosa cells can pass through the zona pellucida, forming a gap connection with the cell membrane of the oocyte. The point of contact between these cell membranes provides a channel for the transmission of information and nutrition to the oocyte.

During the preantral follicle phase, follicle-stimulating hormone (FSH), estrogen and androgen receptors appear in granulosa cells, making granulosa cells capable of responding to the corresponding hormones. Five signaling pathways are important for maintaining the function of granulosa cells. WNT2, WNT4, NOTCH2, NOTCH3, Iagged2, IHH, and DHH are expressed in developing granulosa cells (Albert et al., 2002; Minnie et al., 2002; Mark et al., 2005; Xie et al., 2017). The activation of WNT signaling by WNT2 and WNT4 promotes granulosa cell proliferation and increases the expression of β-catenin and its target cells (Figure 1; Boyer et al., 2010; Wang et al., 2010). Insulin can stimulate cell proliferation in cultured granulosa and theca calls (Saltiel and Kahn, 2001). The proliferation of granulosa cells was shown to be dependent on Notch signaling since treatment with a Notch signaling inhibitor decreased the number of granulosa cells (Figure 1; Jing et al., 2017). The expression of Ihh and Dhh in granulosa cells in growing follicles makes them a source of Hh signaling to activate surrounding theca cells (Mark et al., 2005).

These signaling pathways can work synergistically with hormones. For example, the mRNA level of *Wnt2* is increased in cultured granulosa cells (Castanon et al., 2012); furthermore, activation of β-catenin facilitated FSH-mediated effects in ovarian follicular cells, while deletion of β-catenin in mouse granulosa cells resulted in impaired FSH activity (Hernandez Gifford et al., 2009; Hernandez Gifford, 2015). In addition, insulin work synergistically with FSH to promote the differentiation and proliferation of ovarian theca-interstitial cells (Duleba et al., 1997). The vascular system is another factor that influences follicular development due to its role in providing nutrition, oxygen and hormone support. Both the Notch and Hedgehog signaling pathways are involved in the angiogenesis process (Ren et al., 2009, 2012; Xie et al., 2017). Finally, all five signaling pathways involve molecular interactions with FOXO3, a transcription factor that is essential in follicular development. This indicates the core position of FOXO3 during ovarian development.

In conclusion, the classical signaling pathway reviewed in this article consists of multiple complex layers, and what we know about the role of FOXO3 and its related signaling pathways in the regulation of follicular development continues to grow. However, many mechanisms remain to be determined to gain a better understanding of these signaling molecules in folliculogenesis.

## Author Contributions

LL, XS, and YS co-wrote the manuscript. LL drew the picture. ZW co-wrote the manuscript and designed the structure of the manuscript. All authors read and approved the final manuscript.

## Conflict of Interest

The authors declare that the research was conducted in the absence of any commercial or financial relationships that could be construed as a potential conflict of interest.

## Publisher’s Note

All claims expressed in this article are solely those of the authors and do not necessarily represent those of their affiliated organizations, or those of the publisher, the editors and the reviewers. Any product that may be evaluated in this article, or claim that may be made by its manufacturer, is not guaranteed or endorsed by the publisher.

## Abbreviations

CL: corpus luteum
PmF: primordial follicles
GF: Graafian follicles
PrF: primary follicles
SF: secondary follicles
AF: atretic follicles
PCOS: polycystic ovary syndrome
POF: premature ovarian failure
PI3K: phosphatidylinositol 3-kinase
FOXO3: forkhead box O3
IGF-1: insulin-like growth factor
NICD: Notch intracellular domain
FSCs: follicle stem cells
FSH: follicle-stimulating hormone.
